# Supplementary material for: Unveiling the Bioactive Potential of the Invasive Jellyfish Phyllorhiza punctata Through Integrative Transcriptomic and Proteomic Analyses
Source: Biomolecules. 2025 Aug 4;15(8):1121. doi: 10.3390/biom15081121 (PMC12383608; doi:10.3390/biom15081121)
Supplement: Supplementary file 1 [file biomolecules-15-01121-s001.zip › Suplementary Material/FileS3/PEPFOLD4/XP_065058610_14196/clusters.html]

 PEP-FOLD clusters


Download archive of all models  here   

|  |  |  |
| --- | --- | --- |
| # | file | sOPEP |
| # cluster 1 - Size: 25 |  |  |
|  | XP\_065058610\_14196-model1 | -20.4536 |
|  | XP\_065058610\_14196-model1.1 | -20.3867 |
|  | XP\_065058610\_14196-model1.2 | -20.3411 |
|  | XP\_065058610\_14196-model1.3 | -20.2924 |
|  | XP\_065058610\_14196-model1.4 | -20.2669 |
|  | XP\_065058610\_14196-model1.5 | -20.2576 |
|  | XP\_065058610\_14196-model1.6 | -20.2576 |
|  | XP\_065058610\_14196-model1.7 | -20.2576 |
|  | XP\_065058610\_14196-model1.8 | -20.2199 |
|  | XP\_065058610\_14196-model1.9 | -20.2108 |
|  | XP\_065058610\_14196-model1.10 | -20.2041 |
|  | XP\_065058610\_14196-model1.11 | -20.1869 |
|  | XP\_065058610\_14196-model1.12 | -20.1646 |
|  | XP\_065058610\_14196-model1.13 | -20.1185 |
|  | XP\_065058610\_14196-model1.14 | -19.9643 |
|  | XP\_065058610\_14196-model1.15 | -19.8849 |
|  | XP\_065058610\_14196-model1.16 | -19.8434 |
|  | XP\_065058610\_14196-model1.17 | -19.8322 |
|  | XP\_065058610\_14196-model1.18 | -19.8322 |
|  | XP\_065058610\_14196-model1.19 | -19.7624 |
|  | XP\_065058610\_14196-model1.20 | -19.7057 |
|  | XP\_065058610\_14196-model1.21 | -19.6884 |
|  | XP\_065058610\_14196-model1.22 | -19.6518 |
|  | XP\_065058610\_14196-model1.23 | -19.5574 |
|  | XP\_065058610\_14196-model1.24 | -19.4889 |
| # cluster 2 - Size: 6 |  |  |
|  | XP\_065058610\_14196-model2 | -19.8376 |
|  | XP\_065058610\_14196-model2.1 | -19.4583 |
|  | XP\_065058610\_14196-model2.2 | -19.4295 |
|  | XP\_065058610\_14196-model2.3 | -19.3727 |
|  | XP\_065058610\_14196-model2.4 | -19.1663 |
|  | XP\_065058610\_14196-model2.5 | -17.1268 |
| # cluster 3 - Size: 1 |  |  |
|  | XP\_065058610\_14196-model3 | -17.8054 |
| # cluster 4 - Size: 4 |  |  |
|  | XP\_065058610\_14196-model4 | -14.1181 |
|  | XP\_065058610\_14196-model4.1 | -14.091 |
|  | XP\_065058610\_14196-model4.2 | -13.1514 |
|  | XP\_065058610\_14196-model4.3 | -12.6689 |
| # cluster 5 - Size: 1 |  |  |
|  | XP\_065058610\_14196-model5 | -13.446 |
| # cluster 6 - Size: 2 |  |  |
|  | XP\_065058610\_14196-model6 | -13.1414 |
|  | XP\_065058610\_14196-model6.1 | -13.1198 |
| # cluster 7 - Size: 7 |  |  |
|  | XP\_065058610\_14196-model7 | -12.8049 |
|  | XP\_065058610\_14196-model7.1 | -12.6449 |
|  | XP\_065058610\_14196-model7.2 | -12.4832 |
|  | XP\_065058610\_14196-model7.3 | -12.4104 |
|  | XP\_065058610\_14196-model7.4 | -12.309 |
|  | XP\_065058610\_14196-model7.5 | -12.2428 |
|  | XP\_065058610\_14196-model7.6 | -12.2204 |
| # cluster 8 - Size: 10 |  |  |
|  | XP\_065058610\_14196-model8 | -12.7718 |
|  | XP\_065058610\_14196-model8.1 | -12.6735 |
|  | XP\_065058610\_14196-model8.2 | -12.6502 |
|  | XP\_065058610\_14196-model8.3 | -12.6466 |
|  | XP\_065058610\_14196-model8.4 | -12.5839 |
|  | XP\_065058610\_14196-model8.5 | -12.5665 |
|  | XP\_065058610\_14196-model8.6 | -12.4983 |
|  | XP\_065058610\_14196-model8.7 | -12.457 |
|  | XP\_065058610\_14196-model8.8 | -12.4344 |
|  | XP\_065058610\_14196-model8.9 | -12.3639 |
| # cluster 9 - Size: 2 |  |  |
|  | XP\_065058610\_14196-model9 | -12.3436 |
|  | XP\_065058610\_14196-model9.1 | -12.2979 |
| # cluster 10 - Size: 2 |  |  |
|  | XP\_065058610\_14196-model10 | -9.74332 |
|  | XP\_065058610\_14196-model10.1 | -8.08786 |
| # cluster 11 - Size: 3 |  |  |
|  | XP\_065058610\_14196-model11 | -8.70952 |
|  | XP\_065058610\_14196-model11.1 | -8.48233 |
|  | XP\_065058610\_14196-model11.2 | -8.24977 |
| # cluster 12 - Size: 4 |  |  |
|  | XP\_065058610\_14196-model12 | -8.52682 |
|  | XP\_065058610\_14196-model12.1 | -8.33222 |
|  | XP\_065058610\_14196-model12.2 | -8.07277 |
|  | XP\_065058610\_14196-model12.3 | -7.27898 |
| # cluster 13 - Size: 1 |  |  |
|  | XP\_065058610\_14196-model13 | -8.41741 |
| # cluster 14 - Size: 4 |  |  |
|  | XP\_065058610\_14196-model14 | -7.93923 |
|  | XP\_065058610\_14196-model14.1 | -7.76084 |
|  | XP\_065058610\_14196-model14.2 | -7.46905 |
|  | XP\_065058610\_14196-model14.3 | -6.14738 |
| # cluster 15 - Size: 5 |  |  |
|  | XP\_065058610\_14196-model15 | -7.86066 |
|  | XP\_065058610\_14196-model15.1 | -7.81805 |
|  | XP\_065058610\_14196-model15.2 | -7.76006 |
|  | XP\_065058610\_14196-model15.3 | -7.65457 |
|  | XP\_065058610\_14196-model15.4 | -7.28025 |
| # cluster 16 - Size: 2 |  |  |
|  | XP\_065058610\_14196-model16 | -7.61873 |
|  | XP\_065058610\_14196-model16.1 | -7.04068 |
| # cluster 17 - Size: 2 |  |  |
|  | XP\_065058610\_14196-model17 | -7.58911 |
|  | XP\_065058610\_14196-model17.1 | -6.55084 |
| # cluster 18 - Size: 1 |  |  |
|  | XP\_065058610\_14196-model18 | -7.56107 |
| # cluster 19 - Size: 1 |  |  |
|  | XP\_065058610\_14196-model19 | -7.25039 |
| # cluster 20 - Size: 1 |  |  |
|  | XP\_065058610\_14196-model20 | -7.21297 |
| # cluster 21 - Size: 1 |  |  |
|  | XP\_065058610\_14196-model21 | -7.19255 |
| # cluster 22 - Size: 1 |  |  |
|  | XP\_065058610\_14196-model22 | -7.03792 |
| # cluster 23 - Size: 1 |  |  |
|  | XP\_065058610\_14196-model23 | -7.02812 |
| # cluster 24 - Size: 1 |  |  |
|  | XP\_065058610\_14196-model24 | -6.92447 |
| # cluster 25 - Size: 1 |  |  |
|  | XP\_065058610\_14196-model25 | -6.83901 |
| # cluster 26 - Size: 3 |  |  |
|  | XP\_065058610\_14196-model26 | -6.81671 |
|  | XP\_065058610\_14196-model26.1 | -6.60837 |
|  | XP\_065058610\_14196-model26.2 | -5.93685 |
| # cluster 27 - Size: 1 |  |  |
|  | XP\_065058610\_14196-model27 | -6.55188 |
| # cluster 28 - Size: 1 |  |  |
|  | XP\_065058610\_14196-model28 | -6.40814 |
| # cluster 29 - Size: 2 |  |  |
|  | XP\_065058610\_14196-model29 | -6.2907 |
|  | XP\_065058610\_14196-model29.1 | -6.1463 |
| # cluster 30 - Size: 1 |  |  |
|  | XP\_065058610\_14196-model30 | -6.26711 |
| # cluster 31 - Size: 1 |  |  |
|  | XP\_065058610\_14196-model31 | -5.83983 |
| # cluster 32 - Size: 1 |  |  |
|  | XP\_065058610\_14196-model32 | -5.59471 |
| # cluster 33 - Size: 1 |  |  |
|  | XP\_065058610\_14196-model33 | -5.53784 |
|  |  |  |
